# LIFE EXPECTANCY AND COVID-19 MORTALITY DISPARITIES AMONG NATIVE AMERICAN OLDER ADULTS IN WYOMING

**DOI:** 10.1093/geroni/igae098.3451

**Published:** 2024-12-31

**Authors:** Megan Siebecker, Josephine Boateng, Alexa Fleet, Taylor Jansen, Elizabeth Dugan

**Affiliations:** University of Massachusetts Boston, Boston, Massachusetts, United States; University of Massachusetts Boston, Dorchester, Massachusetts, United States; University of Massachusetts Boston-- Gerontology, Boston, Massachusetts, United States; University of Massachusetts Boston, Boston, Massachusetts, United States; University of Massachusetts Boston, Boston, Massachusetts, United States

## Abstract

Accumulated inequities contribute to health disparities. Striking evidence of this is found in Native older adults in Wyoming. The history of violence and racism against Native peoples in the US is a national shame. Documenting the health challenges experienced by Native older adults brings awareness and potential remedy to this racist history. American Indian and Alaska Native communities are a historically under-researched population and are at greater risk than non-Hispanic white older adults for many chronic conditions. The current study reports on results from the Wyoming Healthy Aging Data Reports (healthyagingdatareports.org). We compared community rates of life expectancy and COVID-19 mortality in Wyoming and in Fremont County (home to the largest proportion of older Native adults in WY). Data sources include US Census Bureau American Community Survey 2016-2020 (population characteristics), Robert Wood Johnson Foundation (community life expectancy), and the Wyoming Department of Health (COVID-19 infection and mortality rates). Small area estimation techniques were used to calculate rates, and ArcGIS software was used for mapping and geographic analyses. Results show an estimated 2,430 Native American older adults who are 55+ are living in Wyoming, and 56% of them reside in Fremont County. Life expectancy rates in Fremont County (73) are 5 years lower than the state average (78.1). There is also a 1.5x higher COVID mortality rate in Fremont County (513.7) as opposed to the state average (340.4). Communities that have experienced systematic discrimination and inadequate health care services have poor outcomes. Policy interventions to address such inequities are warranted.

